# A Single, One-Off Measure of Depression and Anxiety Predicts Future Symptoms, Higher Healthcare Costs, and Lower Quality of Life in Coronary Heart Disease Patients: Analysis from a Multi-Wave, Primary Care Cohort Study

**DOI:** 10.1371/journal.pone.0158163

**Published:** 2016-07-27

**Authors:** Jorge E. Palacios, Mizanur Khondoker, Evanthia Achilla, Andre Tylee, Matthew Hotopf

**Affiliations:** 1 Psychological Medicine Department, Institute of Psychiatry, Psychology, and Neuroscience, King’s College London, London, United Kingdom; 2 NIHR Biomedical Research Centre for Mental Health at the South London and Maudsley NHS Foundation Trust, King’s College London, London, United Kingdom; 3 Department of Applied Health Research, University College London, London, United Kingdom; 4 Health Service and Population Research Department, Institute of Psychiatry, Psychology, and Neuroscience, King’s College London, London, United Kingdom; Chiba University Center for Forensic Mental Health, JAPAN

## Abstract

**Objective:**

To determine whether a one-off, baseline measure of depression and anxiety in a primary care, coronary heart disease (CHD) population predicts ongoing symptoms, costs, and quality of life across a 3-year follow-up.

**Design:**

Longitudinal cohort study.

**Setting:**

16 General Practice surgeries across South-East London

**Participants:**

803 adults (70% male, mean age 71 years) contributing up to 7 follow-up points.

**Main outcome measures:**

Ongoing reporting of symptoms, health care costs, and quality of life.

**Results:**

At baseline, 27% of the sample screened positive for symptoms of depression and anxiety, as measured by the Hospital Anxiety and Depression Scale (HADS). The probability of scoring above the cut-off throughout the follow-up was 71.5% (p<0.001) for those screening positive at baseline, and for those screening negative, the probability of scoring below the cut-off throughout the follow-up was 97.6% (p<0.001). Total health care costs were 39% higher during follow-up for those screening positive (p<0.05). Quality of life as measured by the SF-12 was lower on the mental component during follow-up for those screening positive (-0.75, CI -1.53 to 0.03, p = 0.059), and significantly lower on the physical component (-4.99, CI -6.23 to -.376, p<0.001).

**Conclusions:**

A one-off measure for depression and anxiety symptoms in CHD predicts future symptoms, costs, and quality of life over the subsequent three-years. These findings suggest symptoms of depression and anxiety in CHD persist throughout long periods and are detrimental to a patient’s quality of life, whilst incurring higher health care costs for primary and secondary care services. Screening for these symptoms at the primary care level is important to identify and manage patients at risk of the negative effects of this comorbidity. Implementation of screening, and possible collaborative care strategies and interventions that help mitigate this risk should be the ongoing focus of researchers and policy-makers.

## Introduction

Depression and anxiety symptoms are common in Coronary Heart Disease (CHD) [1] [2]. It is both a causal factor [3,4] and poor prognostic indicator [5], being associated with a range of adverse outcomes, including mortality [6,7], but mechanisms for such associations are incompletely understood [8]. Management strategies in patients with comorbid mood disorders and CHD are problematic [9], partly because of the overlap of symptoms of depression and anxiety with the symptomatology of long-term heart conditions [10]. Randomised controlled trials indicate that antidepressants are effective in improving mood in individuals with depression and CHD [11,12], but have not reduced adverse cardiac outcomes and mortality [13]. There is as yet inconclusive information regarding the dynamic interaction between symptoms of depression and anxiety and how they correlate to CHD and its progression.

Screening for depression in CHD has been controversial since its implementation. Citing the high prevalence of depression in CHD, routine screening for depression in this patient population was recommended by the American Heart Association (AHA) in 2008 [14], but this was challenged shortly after, pointing to a lack of evidence that screening improved outcomes [15]. In the UK, screening for depression was adopted on the Quality and Outcomes Framework (QOF) from 2006 [16], but has since been dropped. NICE guidelines do not recommend screening, although GPs are advised to be alert to depression in at-risk patients (with previous history of mental illness or chronic physical conditions) [17]. There indeed may be insufficient evidence from RCTs to support the recommendation of screening in CHD [18], however there is evidence that screening in conjunction with active management of depression in CHD and diabetes by means of collaborative care may be associated with improved physical and mental health outcomes [19].

One disadvantage of screening is the generation of multiple false positives–i.e. individuals who have transient distress. There is a shortage of longitudinal analysis than can accurately assess the persistence symptoms of depression and anxiety in the course of CHD, and whether an initial screen for these symptoms can predict persistence. Screening might be informative as the comorbidity between CHD and mental disorders increases readmission rates, and overall and outpatient healthcare costs [20], and screening could help to identify and diminish these increased costs. Furthermore, health-related quality of life (QOL) is reduced in CHD patients who have comorbid mental disorders [21], and it is not known whether a single screening tool could help identify those at risk for lower quality of life.

With this in mind, we explore the extent to which a one-off, baseline measure for depression and anxiety symptomatology is predictive of future outcome in a cohort of patients with CHD. Our aims were threefold: Firstly, we aimed to determine the stability of a single positive screen for depression and anxiety. Secondly, we aimed to determine the difference in healthcare costs between those positive and those negative at baseline. Thirdly, we aimed to analyse the differences in quality of life among those measuring positive and those measuring negative. By meeting these objectives we answer how much predictive information a one-off measure of depression and anxiety in CHD patients can provide, an important matter when considering the potential of this measure as a future screening tool in these patients.

## Methods

The population used for this study is derived from the cohort conducted by the UPBEAT UK research programme [22]. This cohort consists of participants identified and recruited from CHD registers in 17 general practices in South London. The sampling frame consisted of 2,938 total number of CHD register patients amongst these 16 practices. These patients were invited to participate by their local GP, of which 917 agreed to be contacted. Of those contacted, 803 (87.6%) agreed to participate and were recruited into the study, and comprises the population we used for these analyses.

Each participant had an initial baseline interview which included the Clinical Interview Schedule Revised (CIS-R) [23], Hospital Anxiety and Depression Scale (HADS) [24], and Patient Health Questionnaire (PHQ-9) [25] to identify depression and anxiety symptoms. They then had follow-up assessments using the HADS and PHQ-9 at 6, 12, 18, 24, 30, and 36 months, done by phone interviews. Each participant therefore had up to 7 time points recorded for symptoms of depression and anxiety. Costs were measured using service use data from the Client Service Receipt Inventory (CSRI) [26], and quality of life data was gathered using the 12-item Short-Form Health Survey (SF-12) [27], both also applied at 6 month intervals.

For the purpose of this analysis, we tested whether a positive result on the HADS at initial assessment was a useful predictor of future reporting of symptoms, costs, and quality of life, and thus a potentially reliable tool for screening patients in this population. The HADS has previously been reported to be a valid screening measure in cardiac patients [28]. Here, we used the HADS total score—comprised of the sum of the depression (HADS-D) and anxiety (HADS-A) subscales—as a measure of general distress. Recently HADS has been argued to be better seen as a general measure of distress in cardiac patients, due in part to the overlapping symptoms of depression and anxiety that make a separation into subscales unreliable [29].

To determine the best scale and cut-off to be used for screening purposes, we first ran a sensitivity analysis comparing the results of the HADS at baseline to those of the CIS-R, to test whether the HADS was an accurate measure of depression/anxiety in our patient population. We calculated the specificity and sensitivity of the HADS with reference to the CIS-R using a Receiver Operating Characteristic (ROC) curve analysis, and thereafter determined the optimal threshold using the ‘point of curve closest to the (0,1)’ criteria [30]. By calculating the distance of each cut-off point on the HADS to the optimal (0,1) point on the graph, we could determine the cut-off point which gave the best combination of sensitivity and specificity. The formula used for calculating the distance was *d*^*2*^ *= [(1–Sn)*^*2*^ *+ (1 –Sp)*^*2*^*]*, where Sn = sensitivity and Sp = specificity [30].

Having determined the cut-off for the HADS as our measure for general distress, we proceeded with the analysis in accordance with our study aims. We tested the initial baseline measurement of the HADS to assess its predictive accuracy across three domains: 1) as a predictor of ongoing symptoms of depression and anxiety; 2) as a predictor of healthcare costs; and 3) as a predictor of quality of life. All analyses were conducted using Stata version 11.2.

### Predicting ongoing symptoms of depression and anxiety

In these analyses we tested the predictive ability of the baseline screen in predicting future screening results on the same measure. We used a random effects logistic regression model for this purpose, clustered by participant ID. The logistic regression framework enabled us to adjust for the effect of time by including it as a covariate in the model. Our predictor variable was the HADS score at baseline, coded as binary, with a value of 1 if the HADS score was at or above the cut-off, and with a value 0 if the score was below. Our outcome variable was the same HADS binary variable measured repeatedly at subsequent follow-up visits, from 6 months to 36 months. This random effects logistic regression model, estimated using maximum likelihood under missing at random (MAR) assumption, also protects against possible bias in the estimated parameters due to missing data. Our model, therefore, shows how strongly baseline ‘caseness’ predicts future ‘caseness’, and concurrently how baseline ‘non-caseness’ predicts future ‘non-caseness’. This is a similar concept to the one describing positive and negative predictive values, where the aim is to determine what percentage of measured cases are true cases (PPV) according to a gold standard, and what percentage of measured non-cases are true non-cases (NPV). Here, our aim is to determine how strongly the reporting of symptoms of depression and anxiety at baseline screen can predict the future reporting of these same symptoms. The results can be interpreted as PPV and NPV for the baseline screening if the follow-up reporting of caseness / non-caseness is treated as the “gold standard”.

For comparison, we also used the same model with the PHQ-9 scale for depression, testing its ability to predict depression from an initial baseline measure. The cut-off used for the PHQ-9 was 10, as is the most widely used and recommended [31], and a recent meta-analysis validated it by showing no significant differences in sensitivity and specificity for cut-off points between 8–11 [32]. We also tested the model on the subscales of the HADS: both the HADS-D and HADS-A, using the most widely used cut-off of 8+ [33] to compare the predictive values of these subscales to the overall score.

We also compared the predictive value of reporting ‘caseness’ and ‘non-caseness’ from a single, one-off baseline measure, to the predictive value of two consecutive measures (baseline and 6 month follow-up). This was with the aim to test whether a single measure is enough to predict continued reporting of symptoms, or if two consecutive measures are needed to more accurately make that prediction.

Finally, as a sensitivity analysis, we ran the models removing those patients who self-reported at baseline as being under current treatment for depression (n = 65), to test whether this had any effect on the results.

### Predicting healthcare costs

For the cost analysis, we gathered the data compiled by the CSRI and ran a t-test to determine the difference in mean costs of patients across the 3-year follow-up between the positive and negative baseline measure groups. These included hospital costs (inpatient, outpatient, A&E) and community costs (GP, district nurse), which add up to the total healthcare costs. Informal costs (assistance provided by family members or carers in day-to-day activities) were also considered, and adding these to the healthcare costs made up the total societal costs. We then conducted a generalised linear regression analysis to explore the predictors for total health care costs. This model was constructed by using costs as the dependent variable, and baseline HADS as the predictive variable, along with age, sex, and ethnicity as covariates. Unit cost estimates were obtained from the Unit Cost of Health and Social Care 2012 [34] and the NHS Reference Costs 2011–12 publications [35]. All costs are reported in Pound Sterling (£).

### Predicting quality of life

For the QOL analysis, we used the SF-12, and calculated both the physical (PCS) and mental (MCS) subscores. The PCS uses the bodily pain, physical functioning, and role (physical) components of the SF-12, whilst the MCS uses the social functioning, mental health, and role (emotional) components. Both incorporate the general health and vitality components to calculate the final score. We then constructed a linear regression model, using the baseline HADS (cut-off 12) binary variable as the predictor variable, and SF-12 as the outcome variable, and controlled for baseline QOL score and time. We then ran the model using sex and age as covariates to test whether these had any effect on the relationship between quality of life and baseline distress.

## Results

### Characteristics of study sample

Full characteristics of the cohort have been published elsewhere [36]. In essence the population is representative of the wider CHD population with a mean age of 70.6 years, a proportion (69.9%) of males. It is largely of white British ethnicity (87.3%). Most individuals in the cohort are retired (77.7%), and live with their husband/wife/partner (61.0%) in an owned home (67.9%). 44.3% reported chest pain at baseline measurement, whilst 42.2% had a documented myocardial infarction (MI). Most common comorbidities included hypertension (55.4%), diabetes (24.9%), renal disease (18.9%), and cancer (12.0%). Around three-quarters of the cohort were overweight or obese (76.0%). 57.3% were an ex-smoker, whilst 29.9% had never smoked.

**Fig 1:**
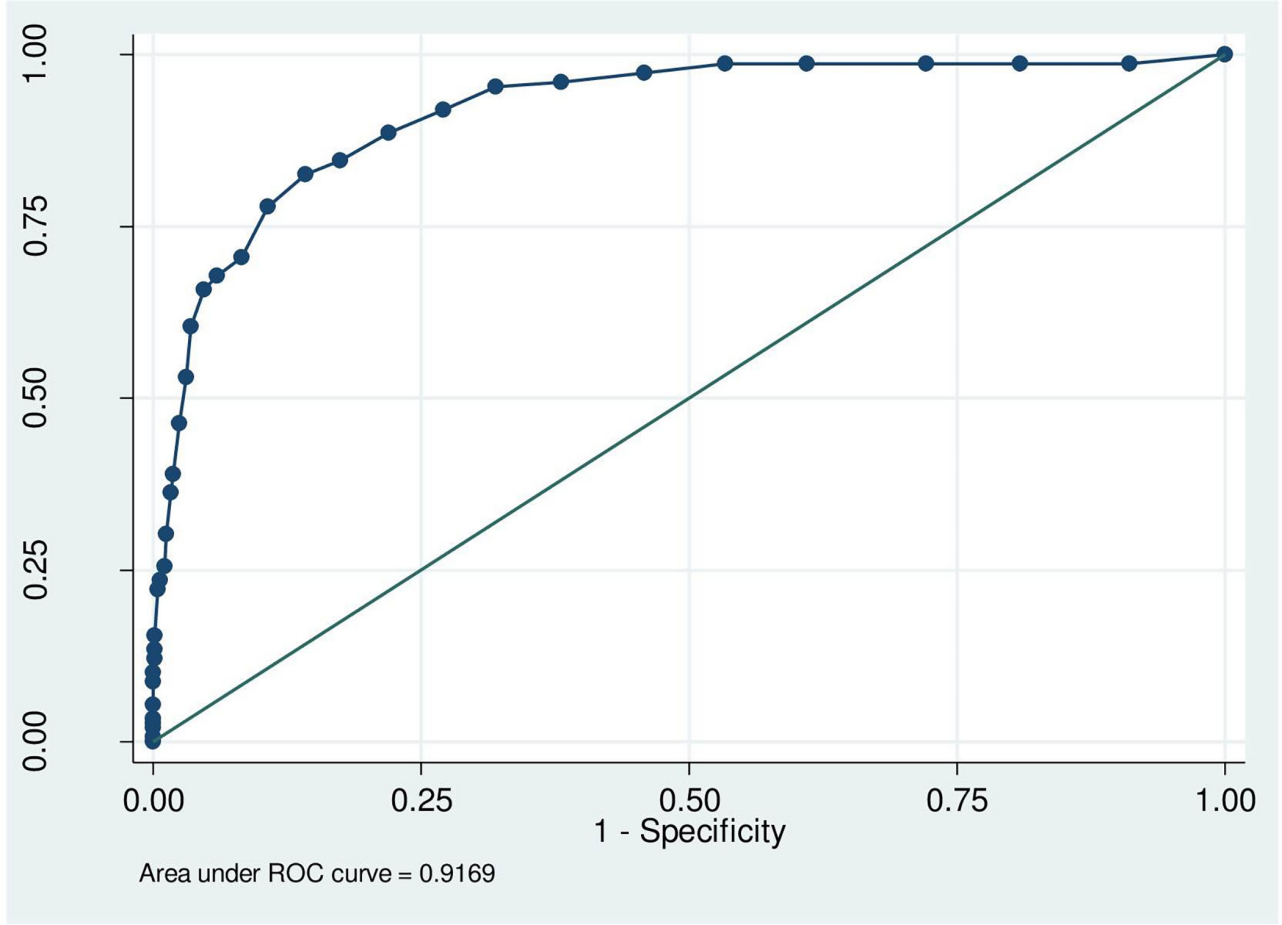
ROC curve analysis measuring predictive accuracy of HADS at baseline with CIS-R based diagnosis as the ‘gold standard’. ROC: Receiver Operating Characteristic.

The results of the sensitivity analyses are summarised in Fig 1 and Table 1. A cut-off of 12 gave the best combination of sensitivity (85.78%) and specificity (82.55%) when measured against any diagnosis of depression and anxiety from the CIS-R, and thus we concluded this was the optimal cut-off point to test for the predictive validity of the HADS in this population.

**Table 1:** Sensitivity, specificity, and distance to (0,1) point of each cut-off point on the HADS.

Cut-off point	Sensitivity (%)	Specificity (%)	Distance to (0,1)
(> = 8)	95.30	68.04	.323
(> = 9)	91.95	72.94	.282
(> = 10)	88.59	77.98	.248
(> = 11)	84.56	82.57	.233
**(> = 12)**	**82.55**	**85.78**	**.225**
(> = 13)	77.85	89.30	.246
(> = 14)	70.47	91.74	.307
(> = 15)	67.79	94.04	.328
(> = 16)	65.77	95.26	.346

Depression and anxiety measures of the sample at baseline were the following: HADS total score had a mean of 8.3 (95% CI: 7.8–8.8) with 26.9% reporting scores at or above 12. 18.6% had some form of disorder according to the CIS-R, with mixed anxiety and depression as the most common (8.2%). The mean depression score on the HADS-D was 3.34, whilst 12.8% had a score above 8 (the accepted cut-off for both subscales of the HADS). The mean anxiety score on the HADS-A was 4.97, with 24.8% of the sample scoring above 8. The PHQ-9 scores were above the cut-off for depression in 15.8% of cases, with a mean of 4.64 (95% CI: 4.27–5.02)

### Reporting of symptoms of depression/anxiety throughout follow-up period

Table 2 shows the proportions of patients with positive symptoms of distress at each time point. The positive scores across the entire cohort ranged from 23.1% (at 12 months) to 27.7% (at 24 and 36 months). Females (range of 29.7–37.8%) reported symptoms of distress more frequently than males (range of 19.9–24.3%), whilst non-white ethnicity (range of 35.4–41.2%) reported higher symptoms compared to whites (range of 21.0–26.5%).

**Table 2:** Reporting of symptoms of distress (HADS with a cut-off of 12+) across time.

	Baseline (n = 803)	6 months (n = 731)	12 months (n = 710)	18 months (n = 668)	24 months (n = 632)	30 months (n = 597)	36 months (n = 573)
***Total sample (%)***	***26*.*9***	***24*.*3***	***23*.*1***	***25*.*6***	***27*.*7***	***27*.*1***	***27*.*7***
**Males (%)**	22.5	19.9	20.3	22.5	24.3	23.8	23.7
**Females (%)**	37.2	34.7	29.7	33.3	35.9	35.0	37.8
**Whites (%)**	24.8	22.6	21.0	24.3	26.5	26.1	26.3
**Non-whites (%)**	41.2	38.1	38.5	35.4	37.1	35.9	40.7

### Model predicting future reporting of symptoms according to baseline measure

The main model is shown in Table 3. It reports that those who have a positive screen at baseline according to the HADS, with a cut-off of 12+, have a 71.5% probability of continuing to report symptoms of distress across the follow-up period of 3 years (labelled as the ‘PPV’ on the table). Conversely, those who have a negative screen at baseline have a 97.6% probability of not reporting these symptoms across the 3 years (labelled as the ‘NPV’ on the table). We also applied the model to the subscales of HADS for symptoms of depression and anxiety. Using the HADS-D, the probability of continued reporting of positive symptoms after a baseline positive screen (cut-off of 8+) was 61.2%, and the probability of non-reporting of symptoms after a baseline negative screen was 98.8%. For the HADS-A, the probability of positive symptoms of anxiety after a baseline positive screen (cut-off of 8+) was 70.0%, and of negative symptoms after a baseline negative screen was 98.3%. Finally, we used the model with the PHQ-9 to compare the results, and found the PHQ-9 to predict a 57.7% probability of continued positive symptoms after a baseline positive screen, and a 98.3% probability of continued negative symptoms after a baseline negative screen. The sensitivity analysis removing the 65 patients who reported being under current treatment for depression did not show any significant differences: the PPV was 67.3% and the NPV was 97.9%.

**Table 3:** Model predicting future reporting of symptoms according to baseline measure.

		PPV (95% CI)	*P value*	NPV (95% CI)	*P value*
**Baseline measure**					
**Distress**	HADS (cutoff 12+)	71.50 (61.96–81.04)	<0.001	97.63 (96.60–98.65)	<0.001
**Depression**	HADS-D (8+)	61.19 (45.90–76.48)	<0.001	98.79 (98.19–99.41)	<0.001
	PHQ-9	57.66 (42.22–71.11)	<0.001	98.35 (94.17–97.20)	<0.001
**Anxiety**	HADS-A (8+)	70.04 (59.49–80.48)	<0.001	98.34 (97.54–99.15)	<0.001

HADS: Hospital Anxiety and Depression Scale, PHQ-9: Patient Health Questionnaire, PPV: Positive Predictive Value, NPV: Negative Predictive Value.

### Model using two consecutive measures of distress

When using both the baseline and 6-month measures of distress on the HADS, we found that for those with two consecutive positive screens (meaning a score at or above 12 at baseline and then again at 6 months), there was an 86.7% probability of continuing reporting of distress above the cut-off of 12 for the rest of the follow-up period. Therefore, two consecutive positive screens are more predictive of future positive screens than a single, baseline screen (86.7 vs 71.5).

### Cost analysis

The results from the cost analysis are shown in Tables 4 and 5. Those scoring at or above 12 on the HADS at baseline had significantly higher costs across all domains. Differences in mean costs per patient were £202 for primary care, £1,398 for secondary care, £90 for informal care, and £1,820 for total healthcare. The total societal cost difference between means was £1,910 per patient. Differences in means between those identified as having any common mental disorder (CMD) on the CISR and those without a CMD were also highly significant, with a total healthcare and total societal cost difference of £3,136 and £3,305 per patient, respectively (Table 3). The generalised linear regression analysis shows that scoring at or above our cut-off of 12 on the HADS at initial assessment significantly increases total health care costs by 39% (p<0.05). Female sex, age, and non-white ethnicity did not significantly increase or decrease total health care costs. The sensitivity analysis did not alter the results; the difference between total societal costs after removing those under self-reported treatment for depression at baseline was £1,630, which was still significant.

**Table 4:** Comparative 3-year mean healthcare and societal costs (2011–12 prices) between groups of patients with positive and negative results on HADS (cut-off 12+) and CISR (any CMD).

Scale	result	n	Primary care	Secondary care	Informal care	Total healthcare	Total societal
**HADS (12+)**	negative	587	£513.53	£3,852.87	£112.67	£4,941.08	£5,053.76
	positive	216	£715.98	£5,250.85	£202.76	£6,761.15	£6,963.92
	*t-test*		*0*.*001*	*0*.*044*	*0*.*011*	*0*.*019*	*0*.*015*
**CISR (Any CMD)**	negative	654	£516.06	£3,808.35	£105.67	£4,848.70	£4,954.37
	positive	149	£795.91	£6,074.84	£274.01	£7,985.08	£8,259.09
	*t-test*		*<0*.*001*	*0*.*004*	*<0*.*001*	*<0*.*001*	*<0*.*001*

HADS: Hospital Anxiety and Depression Scale, CISR: Client Service Receipt Inventory, CMD: Common Mental Disorder.

**Table 5:** Impact on total health care costs of people scoring above the cut-off (12+) on HADS at initial assessment.

Variable	Exp. Coeff. (SE)	95% Conf. Interval
*HADS (12+)*	*1*.*388 (*.*206)*^*1*^	*(1*.*037–1*.*857)*
Sex (Female)	1.086 (.152)	(0.825–1.430)
Age (continuous)	1.002 (.005)	(0.991–1.014)
Ethnicity (non-white)	0.898 (.176)	(0.612–1.319)

^1^ p<0.05.

### Quality of life analysis

Table 6 summarises the results of the linear regression models used to compare QOL scores between people with a positive HADS screen at baseline (cut-off 12+), and people with a negative screen. The first model was adjusted for baseline QOL values and also adjusted for time. Patients with a score of 12+ on the HADS at baseline had a significantly lower score on both subscales of the SF-12 across time in comparison to people with a baseline score below 12. On the mental subcomponent (MCS), the average score was almost one point lower (0.90), whilst on the physical subcomponent (PCS), the average score was more than 4 points lower (4.62). When we adjusted for age and sex, the score on the MCS was still lower (0.75) amongst people with a baseline positive screen for distress, although this was borderline non-significant (p = 0.059). However, the PCS score was almost 5 points lower (4.99), and this remained highly statistically significant. When removing patients with a self-reported treatment for depression at baseline, the results remained significant: the first model showed a PCS of 4.43 points lower, and when adjusting for age and sex the PCS was 4.91.

**Table 6:** Quality of life (QOL) models comparing mean scores across time on MCS and PCS subscales of SF-12, according to baseline HADS (cut-off 12).

Model (covariates)	SF-12 subscore	Coefficient *(95% CI)*	*p value*
**Model 1 (baseline QOL, time)**	MCS	-0.90 (-1.71 to -0.09)	0.029
	PCS	-4.62 (-5.83 to -3.41)	<0.001
**Model 2 (baseline QOL, time, age, sex)**	MCS	-0.75 (-1.53 to 0.03)	0.059
	PCS	-4.99 (-6.23 t-3.76)	<0.001

Model 1 used only baseline score and time as covariates, while Model 2 adjusted for age and sex. SF-12: 12-item Short Form Health Survey, MCS: Mental Component Summary, PCS: Physical Component Summar.

## Discussion

We have demonstrated that a 12-item questionnaire on depression and anxiety symptoms, which is simple and quick to complete in a primary care setting, is a valid measure with an optimal cut-off of 12 in a CHD population. This measure is predictive of future symptoms of distress, healthcare costs, and quality of life.

This study has several strengths. It is the first to report on the effect of a baseline measure for continuing reporting of symptoms of depression and anxiety over a multi-wave follow-up. It also shows how the reporting of these symptoms negatively affects quality of life and increases primary and secondary healthcare costs, using repeated measures across time We achieved satisfactory response rates considering this was done in a primary care setting, constrained with an opt-in recruitment method, and follow-up rates were high throughout the course of the study. Whilst the HADS showed good reliability as compared to the CIS-R at baseline, and we selected the cut-off point with the best overall balance of sensitivity and specificity, we did not have a gold-standard measure to compare HADS to across time. This notwithstanding is counteracted by the fact that we aimed primarily to test whether the results of one baseline measure held firm across time and could predict the persistence of these symptoms, which our data suggests was indeed the case. The study population represents a primary care sample, and whilst patients were recruited directly from the surgeries based solely on an existing diagnosis of CHD, it does not necessarily represent the CHD register as a whole. We also did not remove patients with a positive diagnosis for mood disorders, or under current treatment for depression and/or anxiety from the main analysis, as our data regarding these circumstances was unreliable. However, some authors have suggested removing patients with diagnosis of depression from screening instruments [37], as this would lessen the probability of a selection bias affecting the results, and thus we ran the analysis removing those patients with current treatment for depression, using self-report data. This sensitivity analysis showed no effect on our results on future reporting of symptoms, costs, or quality of life.

Most studies in the existing literature regarding depression and anxiety in the context of CHD use a single assessment and follow-up over time to determine results. We used a multi-wave, repeated measures sample with which we can assess the pattern of reporting of symptoms over time, and have been able to track the results of validated measures of distress, costs, and quality of life, able in this way to accurately assess differences across time between those patients with symptoms of distress at baseline and those without these symptoms.

We echo the recommendations made by previous authors regarding screening for depression in primary care [18], and add that in patients with CHD, anxiety symptoms are equally important and screening should incorporate both. We have shown the HADS to be a reliable predictive measure as a screening tool for symptoms of distress, and to have a cut-off with sufficient sensibility and specificity to identify these symptoms.

This study suggests that the screening of symptoms of depression and anxiety in this population may identify those who are at risk for continuing reporting of these symptoms, and additionally, identify those with a lower quality of life, and those accruing a higher cost to the healthcare system. Furthermore, the HADS is a simple and often-used tool to detect symptoms of depression and anxiety, and we found a score of 12 and above to be a reliable cut-off in this population. This combined depression/anxiety score could perhaps serve better as a screening tool, and we recommend exploring its use further amongst GPs in primary care practice, as well as considering its inclusion in future revisions of the QOF.

We suggest a procedure to dictate future policy in these patients. After a baseline screen, our data suggest that after a negative screen for depression and anxiety symptoms, more follow-ups are not immediately necessary. However, patients who screen positive should be reassessed and followed up for further symptomatology. If they continue to report symptoms at next follow-up, the call is to manage their symptoms actively, rather than risk the effect these symptoms have on negative outcomes.

There have been previous studies arguing for the use of the HADS as a screening instrument in cardiac patients of both sexes [38]. However, there are still questions that remain answering. For example, after screening positive, what is the intervention needed to reduce these symptoms of distress and prevent detrimental disease-specific outcomes and higher mortality risk? This question lies at the core of the suggestion to remove screening for depression in patients with CHD, as there are no reliable management strategies in place after these patients are identified. Indeed, screening only as part of an ‘enhanced care’ package was suggested soon after it was initially implemented in the 2006 QoF [39]. Future research should focus on RCTs that provide interventions for patients who screen positive and analyse the effect on the symptoms themselves, and outcomes of the underlying CHD. A recent pilot study of a personalised care intervention for CHD patients with depression (identified by HADS-D) and current chest pain was found to be feasible and cost-effective in a primary care setting [40].

Ongoing research will analyse the data presented further. For example, the question of causality still remains unanswered, and we will explore whether these symptoms of depression and anxiety are an effect of the underlying physical condition and diminishing quality of life, or the symptoms themselves are worsening the physical condition and lowering the quality of life in these patients. This is especially relevant given the interesting finding that the physical component of the SF-12 has such a strong association to the HADS. We also aim to investigate further into the distinct trajectories of depression and anxiety symptoms that these patients present, and how to interpret those patients which have consistent positive or negative symptoms in comparison to those whose HADS score fluctuates across time.

In the meantime, the identification of concurrent mood disorders in patients with chronic conditions, such as CHD, should be prioritised, in an attempt to turn the tide on the long-term detrimental effects this comorbidity has—both on patients’ lives and the healthcare system as a whole.

## References

[1] Thombs BD, Bass EB, Ford DE, Stewart KJ, Tsilidis KK, Patel U, et al (2006) Prevalence of depression in survivors of acute myocardial infarction. J Gen Intern Med 21: 30–38. 16423120 10.1111/j.1525-1497.2005.00269.x PMC1484630

[2] Tully PJ, Cosh SM, Baumeister H (2014) The anxious heart in whose mind? A systematic review and meta-regression of factors associated with anxiety disorder diagnosis, treatment and morbidity risk in coronary heart disease. J Psychosom Res 77: 439–448. 10.1016/j.jpsychores.2014.10.001 25455809

[3] Hemingway H, Marmot M (1999) Evidence based cardiology: psychosocial factors in the aetiology and prognosis of coronary heart disease. Systematic review of prospective cohort studies. BMJ 318: 1460–1467. 10346775 10.1136/bmj.318.7196.1460 PMC1115843

[4] Lichtman JH, Froelicher ES, Blumenthal JA, Carney RM, Doering LV, Frasure-Smith N, et al (2014) Depression as a risk factor for poor prognosis among patients with acute coronary syndrome: systematic review and recommendations: a scientific statement from the American Heart Association. Circulation 129: 1350–1369. 10.1161/CIR.0000000000000019 24566200

[5] Nicholson A, Kuper H, Hemingway H (2006) Depression as an aetiologic and prognostic factor in coronary heart disease: a meta-analysis of 6362 events among 146 538 participants in 54 observational studies. Eur Heart J 27: 2763–2774. 17082208 10.1093/eurheartj/ehl338

[6] Barth J, Schumacher M, Herrmann-Lingen C (2004) Depression as a risk factor for mortality in patients with coronary heart disease: a meta-analysis. Psychosom Med 66: 802–813. 15564343 10.1097/01.psy.0000146332.53619.b2

[7] Watkins LL, Koch GG, Sherwood A, Blumenthal JA, Davidson JR, O'Connor C, et al (2013) Association of anxiety and depression with all-cause mortality in individuals with coronary heart disease. J Am Heart Assoc 2: e000068 10.1161/JAHA.112.000068 23537805 PMC3647264

[8] Nemeroff CB, Goldschmidt-Clermont PJ (2012) Heartache and heartbreak—the link between depression and cardiovascular disease. Nat Rev Cardiol 9: 526–539. 10.1038/nrcardio.2012.91 22733213

[9] Goldston K, Baillie AJ (2008) Depression and coronary heart disease: a review of the epidemiological evidence, explanatory mechanisms and management approaches. Clin Psychol Rev 28: 288–306. 17601644 10.1016/j.cpr.2007.05.005

[10] Kubzansky LD, Cole SR, Kawachi I, Vokonas P, Sparrow D (2006) Shared and unique contributions of anger, anxiety, and depression to coronary heart disease: a prospective study in the normative aging study. Ann Behav Med 31: 21–29. 16472035 10.1207/s15324796abm3101_5

[11] Berkman LF, Blumenthal J, Burg M, Carney RM, Catellier D, Cowan MJ, et al (2003) Effects of treating depression and low perceived social support on clinical events after myocardial infarction: the Enhancing Recovery in Coronary Heart Disease Patients (ENRICHD) Randomized Trial. JAMA 289: 3106–3116. 12813116 10.1001/jama.289.23.3106

[12] Coventry P, Lovell K, Dickens C, Bower P, Chew-Graham C, McElvenny D, et al (2015) Integrated primary care for patients with mental and physical multimorbidity: cluster randomised controlled trial of collaborative care for patients with depression comorbid with diabetes or cardiovascular disease. BMJ 350: h638 10.1136/bmj.h638 25687344 PMC4353275

[13] Whalley B, Rees K, Davies P, Bennett P, Ebrahim S, Liu Z, et al (2011) Psychological interventions for coronary heart disease. Cochrane Database Syst Rev: CD002902 10.1002/14651858.CD002902.pub3 21833943

[14] Lichtman JH, Bigger JT Jr., Blumenthal JA, Frasure-Smith N, Kaufmann PG, Lesperance F, et al (2008) Depression and coronary heart disease: recommendations for screening, referral, and treatment: a science advisory from the American Heart Association Prevention Committee of the Council on Cardiovascular Nursing, Council on Clinical Cardiology, Council on Epidemiology and Prevention, and Interdisciplinary Council on Quality of Care and Outcomes Research: endorsed by the American Psychiatric Association. Circulation 118: 1768–1775. 10.1161/CIRCULATIONAHA.108.190769 18824640

[15] Ziegelstein RC, Thombs BD, Coyne JC, de Jonge P (2009) Routine screening for depression in patients with coronary heart disease never mind. J Am Coll Cardiol 54: 886–890. 10.1016/j.jacc.2009.01.082 19712796 PMC2749208

[16] BMA & NHS Employers (2006) Revisions to the GMS contract 2006/07. Delivering investment in general practice. BMA, London.

[17] National Institute for Health and Care Excellence (2009) Depression in adults with a chronic physical health problem. Treatment and management. NICE clinical guideline 91. 31886974

[18] Thombs BD, Ziegelstein RC (2014) Does depression screening improve depression outcomes in primary care? BMJ 348: g1253 10.1136/bmj.g1253 24496211

[19] Katon WJ, Lin EH, Von Korff M, Ciechanowski P, Ludman EJ, Young B, et al (2010) Collaborative care for patients with depression and chronic illnesses. N Engl J Med 363: 2611–2620. 10.1056/NEJMoa1003955 21190455 PMC3312811

[20] Baumeister H, Haschke A, Munzinger M, Hutter N, Tully PJ (2015) Inpatient and outpatient costs in patients with coronary artery disease and mental disorders: a systematic review. Biopsychosoc Med 9: 11 10.1186/s13030-015-0039-z 25969694 PMC4427919

[21] Ruo B, Rumsfeld JS, Hlatky MA, Liu H, Browner WS, Whooley MA (2003) Depressive symptoms and health-related quality of life: the Heart and Soul Study. JAMA 290: 215–221. 12851276 10.1001/jama.290.2.215 PMC2776689

[22] Tylee A, Ashworth M, Barley E, Brown J, Chambers J, Farmer A, et al (2011) Up-beat UK: a programme of research into the relationship between coronary heart disease and depression in primary care patients. BMC Fam Pract 12: 38 10.1186/1471-2296-12-38 21605435 PMC3120657

[23] Lewis G, Pelosi AJ, Araya R, Dunn G (1992) Measuring psychiatric disorder in the community: a standardized assessment for use by lay interviewers. Psychol Med 22: 465–486. 1615114 10.1017/s0033291700030415

[24] Zigmond AS, Snaith RP (1983) The hospital anxiety and depression scale. Acta Psychiatr Scand 67: 361–370. 6880820 10.1111/j.1600-0447.1983.tb09716.x

[25] Kroenke K, Spitzer RL, Williams JB (2001) The PHQ-9: validity of a brief depression severity measure. J Gen Intern Med 16: 606–613. 11556941 10.1046/j.1525-1497.2001.016009606.x PMC1495268

[26] Beecham J & Knapp M (2001) Costing psychiatric interventions In: Thornicroft G, editor. Measuring Mental Health Needs. London: Gaskell pp. 220–224.

[27] Ware J Jr., Kosinski M, Keller SD (1996) A 12-Item Short-Form Health Survey: construction of scales and preliminary tests of reliability and validity. Med Care 34: 220–233. 8628042 10.1097/00005650-199603000-00003

[28] Bambauer KZ, Locke SE, Aupont O, Mullan MG, McLaughlin TJ (2005) Using the Hospital Anxiety and Depression Scale to screen for depression in cardiac patients. Gen Hosp Psychiatry 27: 275–284. 15993261 10.1016/j.genhosppsych.2005.03.002

[29] Burns A, Hofer S, Curry P, Sexton E, Doyle F (2014) Revisiting the dimensionality of the Hospital Anxiety and Depression Scale in an international sample of patients with ischaemic heart disease. J Psychosom Res 77: 116–121. 10.1016/j.jpsychores.2014.05.005 25077852

[30] Kumar R, Indrayan A (2011) Receiver operating characteristic (ROC) curve for medical researchers. Indian Pediatr 48: 277–287. 21532099 10.1007/s13312-011-0055-4

[31] Kroenke K, Spitzer RL, Williams JB, Lowe B (2010) The Patient Health Questionnaire Somatic, Anxiety, and Depressive Symptom Scales: a systematic review. Gen Hosp Psychiatry 32: 345–359. 10.1016/j.genhosppsych.2010.03.006 20633738

[32] Manea L, Gilbody S, McMillan D (2012) Optimal cut-off score for diagnosing depression with the Patient Health Questionnaire (PHQ-9): a meta-analysis. CMAJ 184: E191–196. 10.1503/cmaj.110829 22184363 PMC3281183

[33] Bjelland I, Dahl AA, Haug TT, Neckelmann D (2002) The validity of the Hospital Anxiety and Depression Scale. An updated literature review. J Psychosom Res 52: 69–77. 11832252 10.1016/s0022-3999(01)00296-3

[34] Curtis L (2012) Unit Costs of Health and Social Care 2012. Personal Social Service Research Unit, University of Kent.

[35] (2012) NHS Trusts & NHS Foundation Trusts reference cost schedules 2011–12 (NSRC01). In: UK: Department of Health, editor.

[36] Walters P, Barley EA, Mann A, Phillips R, Tylee A (2014) Depression in primary care patients with coronary heart disease: baseline findings from the UPBEAT UK study. PLoS One 9: e98342 10.1371/journal.pone.0098342 24922312 PMC4055485

[37] Thombs BD, Arthurs E, El-Baalbaki G, Meijer A, Ziegelstein RC, Steele RJ (2011) Risk of bias from inclusion of patients who already have diagnosis of. BMJ 343. 10.1136/bmj.d4825 PMC3191850 21852353

[38] Hunt-Shanks T, Blanchard C, Reid R, Fortier M, Cappelli M (2010) A psychometric evaluation of the Hospital Anxiety and Depression Scale in cardiac patients: addressing factor structure and gender invariance. Br J Health Psychol 15: 97–114. 10.1348/135910709X432745 19364446

[39] Gilbody S, Sheldon T, Wessely S (2006) Should we screen for depression? BMJ 332: 1027–1030. 16644833 10.1136/bmj.332.7548.1027 PMC1450055

[40] Barley EA, Walters P, Haddad M, Phillips R, Achilla E, McCrone P, et al (2014) The UPBEAT nurse-delivered personalized care intervention for people with coronary heart disease who report current chest pain and depression: a randomised controlled pilot study. PLoS One 9: e98704 10.1371/journal.pone.0098704 24901956 PMC4047012

